# Practical nursing students’ discursive practices on smoking in Finland

**DOI:** 10.1080/17482631.2019.1610274

**Published:** 2019-05-20

**Authors:** Hanna Aho, Ilkka Pietilä, Katja Joronen

**Affiliations:** a Faculty of Social Science, Health Sciences, University of Tampere, Tampere, Finland; b Department of Musculoskeletal Diseases, Tampere University Hospital, Tampere, Finland; c Faculty of Social Sciences, Social and Public Policy, University of Helsinki, Helsinki, Finland

**Keywords:** Focus group interview, practical nurse, vocational school, views, smoking, Normalization, smoker identity

## Abstract

**Purpose**: Based on focus groups, we analyse how practical nursing students deal with being as smokers and future healthcare workers. The way they justify their smoking is discussed within a group of peers.

**Methods**: The study has a qualitative design with an inductive approach using focus group interviews (FGIs) for data collection. A total of 29 students were interviewed in five groups of five and one group of four participants.

**Results**: In the analysis, we found four different discursive practices the students utilized for rationalizing their own smoking and coping with the moral dilemma of smoking in a context of health care where smoking is forbidden: (1) students normalized smoking with references to its prevalence within their social circles, (2) the students asserted that their smoking was under control, (3) students considered themselves responsible smokers, and (4) students identified smoking as a part of their identity.

**Conclusion**: Training should support the growth of professional identity and address the smoker’s identity right from the start of education. Smokers need special attention in the formulation of professional identity, however, without being stigmatized any further.

## Introduction

Smoking among adolescents is a major public health hazard. Smoking is associated with many health risks such as alcohol and other substance use (O’Loughlin, Dugas, O’Loughlin, Karp, & Sylvestre, 2014) and a lower level of physical activity (Lebron et al., 2017). In addition, there is evidence of those behaviours often occurring simultaneously (Blake, Malik, Mo, & Pisano, 2011) leading to fatal diseases and health inequalities in adulthood (World Health Organization, 2015). Public opinion of desirable behaviour is partly articulated and shaped by health authorities, policies, and legislation around the world. The law on tobacco (2016) in Finland forbids smoking in public buildings and outside areas around schools and institutions where mainly underage (i.e., under 18) people study.

Healthcare professionals who smoke feel obligated to set an example for patients, clients, and visitors, aided by the fact that hospitals and other healthcare environments are smoke-free by law (Law on tobacco, 2016). Therefore, smoking in a healthcare setting is both forbidden and disapproved of. Although rates of smoking have decreased in recent decades, approximately one-fifth of nurses still smoke on a daily basis in the UK (Blake et al., 2011). According to a systematic review of international studies, the prevalence of nurses who smoke ranged from 4.0% to 47.1% and was lower in the studies conducted in North America as compared to the European studies (Duaso, Bakhshi, Mujika, Purssell, & While, 2017). However, according to a study conducted in the US, every fifth practical nurse smoked (21%), which was significantly more than the percentage of doctors (2%) or nurses (7.1%) who smoked. (Sarna, Bialous, Nandy, & Antonio A&Y, 2014)

The stigma of smoking has reportedly caused negative feelings such as guilt, shame, and embarrassment. (Antin, Annechino, Hunt, Lipperman-Kreda, & Young, 2017; Evans-Polce, Castaldelli-Maia, Schomerus, & Evans-Lacko, 2015). The stigma of health-compromising behaviour such as smoking is considered particularly tainting among healthcare workers. Nurses have reported discomfort and fear of patients noticing that they are smokers. (Mujika, Arantzamendi, Lopez‐Dicastillo, Forbes, 2017) There is evidence of student nurses who smoke having even greater knowledge of the health consequences of smoking than non-smoking student nurses do (Sejr & Osler, 2002), but the knowledge does not transfer to the quality of nurse-led health promotion and smoking cessation practices (Duaso et al., 2017). Professional identity develops throughout a nursing career, but nursing education is a key period; during this time students gain the knowledge and skills that separate nurses as professional healthcare workers from lay people (Johnson, Cowin, Wilson, & Young, 2012). The combination of professional duties and their own smoking have been found to cause internal conflicts and to hinder patient education on smoking cessation in registered nurses. Furthermore, leading cessation programs for others is problematic when the leaders of the program have not been able to quit themselves (Mujika et al., 2017).

Practical nursing students are aware of the health problems of smoking and that smoking is considered negative behaviour by the public; moreover, the public views smoking by healthcare workers especially negatively. This places smokers in a socially disadvantaged and morally questionable position, because they work within a healthcare ethos in which smoking is both stigmatizing and can also result in penalties such as warnings, fines, and loss of job for not following smoking restrictions set by employers and the law (Law on tobacco, 2016).

In this study, we analyse, based on interview material from focus groups, how practical nursing students (i.e., nurse assistant students in Finland) deal with their potentially unstable position as smokers and future healthcare workers. How they justify their smoking is discussed within a group of peers.

## Materials and methods

### Ethical considerations

The research plan was approved by the City of Tampere (29 September 2016). Parental consent to participate in the study was not obtained due to local regulations. Approving a research plan at the school management level is considered sufficient to protect students from misconducts.

### Study design

This exploratory qualitative study used a total of 29 volunteers aged 16 to 25 who were interviewed in five focus groups of five and one focus group of four participants. Interviews were conducted by the first author and sessions lasted from 45 to 60 min.

The advantage of focus groups is that they allow access to research participants who might find one-on-one interaction intimidating. (Schulze & Angermeyer, 2003) A focus group allows for complex issues to be explored, yielding richer data than individual interviews would elicit. The group interaction prompts greater depth of information and can lead to greater insight into experiences (Carey & Asbury, 2016); as the group members have the opportunity to hear others’ views, they can also modify, refine, or extend their own contributions. (Ritchie, Ritchie, & Lewis, 2003) We are using extracts from the interviews when reporting findings. Participant names have been numerated to protect the anonymity of the students.

### Participants and data collection

Practical nursing students were recruited from two vocational schools in one city in southern Finland (over 200,000 inhabitants). All participants were female and daily smokers. Practical nursing students were chosen for the target group, as practical nurses will be hired for smoke-free workplaces such as hospitals, home care, and different outpatient and institutional units for the care of the elderly. Smoking during work hours is against the law (Law on tobacco, 2016), employer rules and morally questionable behaviour for social and healthcare workers. Additionally, there is evidence that nurses who smoke might hinder health promotion practices as they may unconsciously belittle negative effects that smoking has on their clients’/patients’ health (Mujika et al., 2017).

First, the researcher visited school classes, introducing the study, and invited all regular smokers to join. However, most of those who agreed to participate in group discussions failed to show up on the day of the interview. To find the smokers, the researcher went to meet them outside in the designated smoking area to introduce the study. The researcher recruited 5 participants from school classes, 3 participants from the corridors, and 12 students from around the ashtray.

Consent forms with permission to tape the discussions and project information sheets were handed out before the group interview. Students were informed of the voluntary nature of the interview and given the opportunity to ask questions prior to the interview. Students were also informed that they could leave the focus group at any time. Participants were given a one-page questionnaire about their family background. The confidential questionnaire was returned by 21 of 29 students (Table 1). Noteworthy was that according to students, there was only one family where neither parent had ever smoked. Ten mothers and five fathers were previous daily smokers but had quit smoking. A recent study conducted with vocational students found that daily parental smoking predicted adolescent daily smoking, and this association was also seen for those adolescents whose parents had quit smoking. (Aho, Koivisto, Paavilainen, & Joronen, 2017) Discussions revealed that three students had a toddler.

All six focus group discussions were audio-recorded, and notes were taken throughout the sessions by the first author. After every interview, the moderator transcribed it verbatim. Laughs, pauses, and expressions were included to transcription. Focus group interviews were held at school premises during school hours, and no fee was paid for interviews. During group interviews, social involvement was the leading theme; parental involvement, school connectedness, positive and negative feelings about smoking, and potential willingness to quit smoking in the future were explored. The themes were introduced by the moderator for students to discuss and share experiences of these wider topics (table 2).

### Method of analysis

This study uses the methodology of critical discourse analysis (CDA). (Fairclough, 2013) CDA can be defined as the analysis of its dialectical relations between discourse and elements or moments as well as internal relations of discourse. Discourse is how we make sense of our society; we develop relationships with others and share meaning through discourse with other people. (De Chesnay, 2014) Discourse analysis is widely used for multiple health issues and research topics such as health promotion, schools’ policy analysis for smoking and alcohol use (Leow, 2011), and smokers’ views of their smoking. (Gough, Fry, Grogan, & Conner, 2009)

For the purposes of our study, we concentrated on how the focus group members dealt with the tensions between two contradictory discourses to analyse the disadvantages and advantages of smoking. In particular, (1) one emphasizing the negative health effects and potential moral condemnation related to practical nursing students’ smoking, and another (2) describing the enjoyable elements and social acceptance of smoking widely prevalent in these students’ social circles. We thus analysed the dialectical nature of how these different discourses emerged in our interviewees’ considerations of their own smoking, and the discursive practices they adopted for both justifying their smoking and coping with potential accusations of health-damaging and irresponsible behaviour. All three authors of this article conducted the analysis by reading the transcripts and listening to audio recordings of the interviews several times to get a preliminary idea of the data. Discussions of similar contents were then started to group together themes. In the analysis, we found four different discursive practices that the students utilized for rationalizing their own smoking and playing down potential moral accusations of acting irresponsibly.

### Findings

In the analysis, we found four different discursive practices the students utilized for rationalizing their smoking as future healthcare workers: (1) smoking was normalized, (2) smoking was claimed to be under control, (3) students considered themselves responsible smokers, and (3) smoking was a part of students’ identity. Moral accusations and the dismay of others did not cause intentions to stop smoking, students were merely annoyed about these attitudes. In their opinions, there was no reason to stop smoking because of their work as patient’s caretaker.

#### Normalization

In focus group discussions, smoking was to a large extent described as a normal behaviour, which was most often justified by the notion that most of the students’ *family members and all close friends smoke*. Normalization refers to social processes through which ideas and actions become “natural” in everyday life and are accepted only among peers in certain settings. (Hathaway, Comeau, & Erickson, 2011; Measham & Shiner, 2009) In students’ discussions, it was evident that family members and friends lowered the threshold to start smoking.

S10: So pretty sure that cos my whole family smoke, my sisters and brothers and everyone else, and when the school and bunch of mates changes, that at least had an effect on me.

S11: I associate it with the most important people and it’s like hanging out together. It’s a pretty uniting factor.

Moderator: You mean friends?

S11: Yes. My friends and quite a lot of my family smoke and so that’s how it went. I started at the end of year 8 (age 14) and just a bit, but then it got out of hand. (laughter)

S12: With me, it started so that there were really a lot of my bunch of mates who smoked now and then and then, out of my family, my mum smokes and more or less my whole family too, and so, it was the friends and then really that I’d give it a go and it was just at the end of year (age 14), to begin with a bit less but then already after a year it became a real habit, like a daily one.

Students discussed how parents reacted when they first were discovered to be smokers in early adolescence. Smoking parents saw smoking as *a normal phase of getting into adolescence*. Some mothers had compared smoking and other substance use as “there is nothing wrong with smoking if you just don’t do drugs”. Also, siblings considered smoking a normal behaviour. The response of many of the parents was to discuss the health and beauty effects of smoking but to let the adolescent make the final decision. Non-smoking parents usually had more comprehensive discussions with their offspring.

One way of normalizing smoking was discussed as *smoking goes well with everything* one does during the day, as if without smoking, walking and eating would not be fulfilling. It was discussed that smoking is a normal routine with every task you do during the day. In addition, smoking attached to moments when it was particularly boring, just like waiting for the bus or walking from the bus to school or home.

S7: Or it’s like, that it fits in with everything. When you wake up and get your first coffee in you, then you always have a fag at the same time and when you walk home from the bus stop, that’s another fag and when you walk a few dozens of metres from the bus stop to the school gates that’s another fag, and then when your stressed after lessons that’s another one each time again, and also when you wait for the bus, and when you take the dogs out for a piss, that’s a fag again, and when you’ve just eaten, you have another one and when you’ve had a shower, you have a fag too. (laughter)

Laughter

*Quitting* smoking might also lead to *exclusion from the smoker group* and friends. That is one reason given for not wanting to quit smoking. S7 was discussing this exclusion, not as peer pressure but more of wanting to be a part of this particular group, and as a non-smoker, it would be awkward.

S7: So it’s like that with me; it could be slightly difficult at this stage. When properly all your mates smoke and when we sit around at ours and play cards, they’re like, send them out for a fag. We can’t really stay alone with those cards.

(laughs)

S7: And then I had to go along with it. It’d get a bit difficult if I started to quit at this stage. But, in a way, I don’t even want to.

All of the discussions included the notion that smoking was normal behaviour given that some teachers are often so irritating. Students claimed having a low tolerance for “crap” from others. Smoking *allows one to calm down* if someone is ignoring your text, or if the noise or stress is getting to be too much to handle. Some teachers were especially difficult to tolerate, causing negative feelings that only smoking could relieve.

S1: It’s like that here at least too, then when we have a few teachers like that you just can’t stand them.

Laughter

S1: You wait through that whole lesson and then get to smoke one cig.

General approval and murmuring

S1: You don’t think about anything else than when you get there to smoke the cig and then you have to hang around to get out for one again.

S4: The last lesson of the day and you see that now I’ve got that teacher and think if I go now and then ask after 45 minutes if I can go to the toilet.

S3: (laughs)

S4: And maybe after that I can cope with this teacher for that 45 minutes, but no more.

Discursive practices for the normalization of smoking not only describe smoking as a general activity in social surroundings but also describe it as a customary way of managing their own emotional states. Boredom was difficult to tolerate. The reason for smoking a cigarette was triggered by monotonous lessons, where students used their boredom to think when they could smoke again instead of trying harder to understand the lesson.

Discussions of smoke-free workplaces, smoking while training, or while working the normalization of smoking was evident: Students pointed out that it would be normal to smoke during the work day since everybody else smokes as well; even the managers smoke or silently give permission to smoke. Smoking during the day was considered not problematic if one did not extend breaks, but on the other hand, it was argued that non-smokers would not get breaks and smoking was considered a way of getting a break during working hours. Smoking tutors were considered valuable, as tutoring around the ashtray created a belonging that could not be obtained otherwise. In other words, one would get better tutoring from a tutor who smoked. While working at hospitals, with the elderly and home health care, smoking was considered normal behaviour that no one could or should do anything about. Daycare centres were considered more as smoke-free workplaces. Smoking was considered by the students to be more acceptable if one could smoke while children were resting during the day, without the children being able to see. When working with the elderly, smoking was considered normal behaviour, as old folks would say if they were affected by the smell of cigarettes.

S25: Nobody actually commented anyway, and there were pretty direct people there. But there’s a routine that you have a smoking coat when you go out to the balcony, smoke a cig, take the coat off, wash your hands and put chewing gum in your mouth, and that already helps a lot. And with us, it was completely necessary to wash your hands when I was in the MRSA department; but you absolutely had to always wash your hands anyway.

Moderator: So S27, have you thought about when even your tutor smelt like…

Laughter

S27: Well, I did think about it, when things went on, like we had a few who were in bed the whole time and they were these two bigger women, so you had to go right in front of their faces (puts the hand close to the face). So sometimes it came up that maybe they don’t really smell it or they don’t want to say, or it just doesn’t really do any harm. But nothing major, just because nobody commented on it and maybe it was also because half the staff actually smoked where I was, and it became a bit like they’re perhaps used to it but there too it was like whenever we came we washed our hands and someone always had lozenges and doled them out to everyone (laughter).

S25: And you have to say that when older people were younger you could smoke everywhere, so could it be a bit to do with that they’ve smelt it everywhere, do you get what I’m trying to say?

S26, S27, S28 [agreeing nodding]

S27: When you’ve smoked in some place indoors, maybe then it doesn’t…

Additionally, an element of normalization was the thought that in the youth of old people, smoking had been more common, and according to the interviewees, *smoking is, therefore, more “normal” to elderly* than to present-day adults. The students did not think about the inequality between themselves and the patient, where the patient is dependent on their caregiver. The smell of cigarettes causing poor appetite or nausea in the elderly was not considered, since the elderly were given the responsibility to say if smoking is a disadvantage for them.

#### Smoking is under control

The negative stereotype of smokers being wrinkly, having yellow teeth, and ill was discussed among the students, but it was not thought of importance, as all the students were adolescents or young adults. In many descriptions, smoking was considered voluntary, as a choice of behaviour that *could be stopped if desired*. Several reasons were given as to why cessation was not considered as favourable right now: school was stressful, personal life outside the school was stressing, and a pause with a cigarette was needed in order to be a better wife, or the student was not motivated or did not find the need for cessation. *Pregnancy in the future was seen as motivation* enough to quit smoking. Students discussed in several focus groups that *quitting smoking while pregnant is easy* as smoking does not give a positive feeling any more when pregnant, and several students had examples of sister or friend quitting while pregnant. This mindset might prevent students of tobacco cessation before becoming pregnant (when cessation is “easier and comes naturally”). Most of the younger participants were sure that they would never start smoking again after pregnancy, but those with children claimed that it was not so easy.

S16: And then thinking that I’ll stop when I’m 25 or something…

S19: I said as well that I’d stop when I was 20; I promised that to myself when I was 16.

Moderator: So why would you stop it?

S16: Cos it doesn’t look that nice if you smoke at 50 or when you’re old so you can just about walk, it really doesn’t look that nice. I’m not saying that it is now either, but the situation is a bit different then…

S18: I’ve smelt some old person’s clothes who smokes tobacco indoors and the smell’s really awful and it’s like you feel sick and I don’t want to be that kind of old woman.

S17: And when everyone, when you get older, starts looking and otherwise seeming bad, then somehow, I don’t want to be stuck with that. Like with many relatives I’ve seen, it starts to show around 40 if you don’t really get a hold of it.

S18: Mostly I’ve been thinking about if at some point you get pregnant, could you stop then?

S17: Yeah, it’s then at the latest.

In the discussions, smoking was seen as a temporary phase of teenage and young adult life, as the interviewees emphasized that smoking and future cessation were under their control. Students systematically decided to quit smoking before a certain age when the adverse effects of smoking begin to emerge. According to longitudinal studies, however, smoking as a teenager predicts smoking as an adult. (Saddleson et al., 2016; Sargent, Gabrielli, Budney, Soneji, & Wills, 2017)

#### A responsible smoker

In all the group discussions students presented themselves as responsible smokers who actively took *non-smokers and children into consideration*. The discussions stated that it is acceptable to smoke, but you must smoke responsibly and take other people into consideration, as they did. In all discussions came up that students would never smoke in front of grandparents, other family members, or relatives who did not approve of smoking. S28 slightly criticized the invisible rules that smokers need to take nonsmokers into consideration, but as no one wanted to admit rebelling against these rules, she continued in the role of a responsible and good-mannered girl. The comment 28 made was accepted by her peers.

Moderator: Do you have some situation where you don’t smoke?

S29: When the grandparents are there or small children close by.

S28: With the other grandparents and when working with children.

Moderator: What if there are totally unfamiliar children at the bus stop?

S28: I just go further away, that’s just the way it is.

S26: So do I.

S28: I don’t go even close or if it happens that I’m there and they come near it’s me who moves; it’s not their responsibility. On the other hand, it’s not my responsibility either because I’m a smart and photogenic person (laughter).

S27: Hmm. [approval]

S28: So of course I go further away from

Protecting children came up several times in the discursive practices, specifically, avoiding smoking with children present. Pregnant mothers and mothers who smoke while pushing a baby buggy were considered not responsible smokers and were discussed with disdain. On the other hand, if smoking could not be quit while pregnant, should mother hide from gazes to smoke?

S24: And I’ve never understood that when you’re in the last stage of pregnancy, why you have to smoke in a public place. Can’t you do it somewhere out of sight!

S21: Hmm.

S24: I at least couldn’t do that if there was a situation where everyone could see it…

S21 and S20: Hmm. Yeah.

Moderator: So perhaps it’s like that it makes the onlooker feel uncomfortable?

[General nodding in agreement and ‘Hmm, yeah’s]

Moderator: There’s a lot of talk these days about passive smoking, so do you smoke, for example with the children there in the car?

S21: No.

S20: No.

S24. No, not ever, even though I don’t have children now, but just generally, if they’re in the car…

S21: Yeah, it’s probably that if there’s a three-year-old child in the back seat and you smoke, you can’t be thinking too straight to do it so passively. It’s a bit different if you smoke outside, the wind takes it away, but in a closed car…some limit comes up, like is the child all right.

Motives to not smoke while around children or being pregnant were ambivalent: partly for the child’s health protection, partly because of the protection from the disgust of other people. Cigarettes could be smoked when concealed from those who disapproved smoking. Students despised pregnant mothers who were not able to quit smoking but understood the difficulty of quitting. Smoking with children in the car was considered irresponsible and did not get any understanding.

#### Defending smoking: smoker identity

Justifying smoking was evident as smoking was considered a part of smokers’ identity, a part of themselves. Smoker identity was considered so dominant that students made it clear that nothing and no one could make them quit smoking: not family nor friends, not partners nor school personnel. Not even getting seriously ill, even developing cancer, was a sufficient reason for quitting. Also, students claimed needing cigarettes was part of their personality. Defending smoking came up in discussions, as smoking is (1) *a connecting family and friends together* and (2) *a medication for blues* and temperamental personality. Justifying smoking was also seen as underrating the cost of cigarettes, and the link between health effects and smoking.

Smoking was discussed as it connects family members and friends together, and new people, even boyfriends, had been found by asking for a cigarette. An idea came up in all focus group discussions that if the students had not smoked, they would never have met the friends they can relate to or been a part of a smoker group where they strongly felt social belonging. In social psychology, the social identification process refers to a situation where, for example, a smoker considered to be a part of a smoker group has a partly unconscious fear of losing this status, leading to exclusion from the group. (Tajfel, 1981)

Moderator: Can you say what benefit there is from smoking?

S14: I met my boyfriend through it, when we went out for a smoke. I wouldn’t have bothered to drag myself out otherwise.

S14: And then I’ve been thinking that when I came to this school and think about what good friends I’ve got, so if you think about those that don’t smoke, they weren’t at all my kind of people.

S11: Yeah, that’s true.

S14: And then I’ve thought that I’d have been in totally the wrong crowd.

All the participants explicitly described their smoking as a positive for social connections that bring people with similar views together. Social belonging to the smoker group was also named when discussions turned to why they would not quit smoking. Furthermore, smoking was given as an opportunity to take a break from stressful situations with children at home.

Moderator: Don’t you have a partner who doesn’t smoke?

S20: Well, I do but it’s like I won’t stop because of him. He’s said a hundred times that I should stop, but I’m not quitting for his sake. I have thought now that I could quit but then I just smoke more.

S23: Yeah.

Moderator: What if your child said that that’s disgusting, stop?

S20: Well, then you go in secret. Probably.

S23: It’s just right now I’ve got no interest or desire to stop smoking. This might sound a bit horrible but when you have a child, then you get a few minutes of time to yourself when you go out into the yard. You get that breather. It sounds stupid, but that’s just how it is. (slightly embarrassed)

Participants discussed defending their smoking to a non-smoking boyfriend, arguing that smokers were well balanced and better girlfriends than if they were non-smokers. Additionally, it was discussed in the group that no one could make the decision of cessation for them. Being asked and pleaded with to quit made interviewees aggrieved, as their smoking was merely a part of their identity and not just a habit. Similarly, young mothers justified their smoking by getting a moment of their own time for a respite.

Smoker identity was discussed as cigarettes were needed to calm difficult personalities or just as medication for anxiety and depression. Without cigarettes, students felt they would lose their identity, their personality would change, and reality would be too much to handle. The group was discussing advantages of smoking in quite a superficial way until S8 began to discuss her need for cigarettes, not because smoking is a fun or nice social habit but needing to smoke as medication for her temperamental personality. This conversation took place after they had just claimed to have their smoking under control and that they could quit if they wanted to.

S8: I can’t think like that when I smoke I’ll probably have cancer. I think about it pretty much like so that I sort of need it. Sure, at some point when I’m able to manage my own emotions so that my nerves don’t really snap in one go. Then I can quit.

Silence

S7: Yeah, it helps me when I get stressed really often and a lot. And just, like, when someone irritates the hell out of you, it’s like that…

S8: It gives you some kind of feeling of pleasure.

S7: Yeah, it relaxes you.

S6: Exactly, it cams you down. And often when I get stressed or annoyed, I just go and sort of calm down with a smoke.

S6: With it, all those bad feelings sort of fade away.

S9: Or like, when mum notices with me. When there was one day that I hadn’t smoked. So, straightaway when I went home my mum, or rather I was like really angry that mum right away was, like: looks like you haven’t smoked. So she sort of notices.

Silence

Moderator: Even though she (the mother) doesn’t want that you smoke, so…

S9: Or, like I don’t know if I lose my temper but I’m really like I cry a lot and have sort of a depressed feeling. (confused murmuring)

S8: Me too, in the summer last year, there were moments where I didn’t have money at all and I was crying all the time and being like I can’t cope, the shakes and everything come, and I start feeling like nothing will work out and the situation ended up with my mum going to get me fags, which she’s never done before. So she, like, got some for me for the first time. She probably thinks pretty much in the same way about it, that I’m much nicer when I have that tobacco. For example, if I don’t have fags, then I don’t come to school, because I can’t spoil anyone else’s day, because I feel so bad. So, it gives you like a feeling of pleasure or something.

In discursive practices, the identity of the smoker was strong. As smokers, they described themselves as being nicer and able to control their negative feelings. Mothers were worried about their daughters’ behaviour if they had not smoked. Likewise, the student thought her mother liked her more as a smoker, even though the mother did not accept smoking.

## Discussion

In this study, we focused on how the practical nursing students in focus groups dealt with their unstable position as smokers in healthcare settings. First, we analysed the dialectical nature of how different discourses emerged in our interviewees’ considerations of their own smoking. Second, we analysed the discursive practices students adopted for both justifying their smoking and coping with potential accusations on their health-damaging and irresponsible behaviour. In the analysis, we found four different discursive practices the students utilized for rationalizing their own smoking and playing down potential moral accusations of acting irresponsibly: (1) smoking was normalized, (2) smoking was claimed to be under control, (3) students considered themselves responsible smokers, and (3) smoking was a part of students’ identity.

In this study, an essential factor in the normalization of smoking was family and friends who smoked. This was important both at the time smoking began and later as a factor preventing cessation. Normalization of smoking refers to smoking considered as normal behaviour, although the reasoning and mindsets would be the opposite of common opinion. This reasoning is understood mainly only by the peers and family that behave the same way. The concept of normalization has been used traditionally in research on drug-related attitudes, but according to Measham et al. (2016), normalization among cigarette smokers has been noted already in the 1990s in the UK. (Measham & Shiner, 2009) A growing cultural stigma towards smoking emerged as a response to the denormalization of the health authorities’ global ban on smoking. (Measham, O’Brien, & Turnbull, 2016) The stigma of smoking is exceptionally tainting in healthcare settings where smoking is legally banned.

The students acknowledged the stigma of smoking and smoking as health-endangering behaviour, but smoking was justified the way that smoking was under their control. As all bad things happen later in life, smoking could be quit later as well. Several reasons were given as to why cessation is not possible during studies. However, longitudinal research evidence confirms that smoking cessation in adolescence is rarely successful (Saddleson et al., 2016; Sargent et al., 2017) and adolescents who were daily smokers at age 12–21 were equally likely to be smokers 14 years later. (Saddleson et al., 2016) Quitting smoking for their work environment now or later in life was not mentioned, but pregnancy was considered the time that smoking needs be quit. Despite good intentions—and some women do quit when they become pregnant—many continue to use tobacco. The World Health Organization (2013) estimates that smoking while pregnant occurs in up to 20% of all mothers giving birth. Noteworthy is that although smoking is declining, the number of smokers during pregnancy has not diminished in the same proportion. (National Institute for Health and Welfare, 2007)

Practical nursing students argued that despite their choice to smoke, they had a responsibility towards non-smokers, particularly children, who needed to be protected from the model of smoking and passive smoking. Not smoking while close relatives and grandparents were present was not protecting them as non-smokers, but merely being protected against the disapproval of people who were special in their lives. Responsibility towards non-smokers was considered a way to justify smoking as not causing harm to non-smokers. Similarity, Evans-Polce et al. (2015) found that some smokers made a distinction between “typical smoker” and themselves to differentiate between a stereotypical smoker and herself and perhaps to emphasize superiority as a smoker (Evans-Polce et al., 2015).

Smoker identity was evident in all discussions. Firstly, smoker identity was discussed as smoothing anxiety and depressive moods. It was claimed that they would be nicer and better able to control negative feelings when they smoked. Even their mother would like them better when they smoked and felt more balanced. Secondly, smoker identity was seen as a way of connecting smoking family and friends together in a meaningful group that separated them from others. Furthermore, new friends and boyfriends had been found by asking for a cigarette, as it was considered a nice way to start talking to strangers. The fear of being excluded from the smokers' group was intense, preventing them from cessation even though their awareness of the disadvantages of smoking was obvious. According to Tajfel (1981), smokers’ personal identity relates to group identity, where the distinction of us and them, smokers and non-smokers, is made. The fear of losing social status leading to being excluded from a group of people with similar attitudes will eventually become a part of self-identity. According to Hall (1971), professional growth in the career sub-identity and career commitment is a series of cycles of challenging goal-setting, independent effort, success, sub-identity growth, and increased career self-esteem and commitment, leading, in turn, to further goal-setting (Hall, 1971). Harré’s theory of the formation of a professional identity holds that professional identity forms through social identity. The theory is a multi-stage process that begins in education and incorporates the adoption of models, values, and beliefs of professionally important communities that the professional manages over time. (Harré, 1983)

For nursing culture, it is important how professional values are learned as well-developed professional identity improves nursing care, healthcare deliveries, and outcomes. (Hunter & Cook, 2018) Professional development and the formation of professional identity are not solely under teachers, curriculum, and policymakers’ responsibility but also part of qualified practical nurses’ and nurses’ duty that is responsible for tutoring students during on-the-job trainings. Tutors are authorities whose views are generally not opposed (Hunter & Cook, 2018) and therefore have a critical influence on how professional identity forms. The language and practices used in practical training support vocational growth and development of the professional identity of a student through the hidden curriculum. Therefore, tutoring can either enhance or negatively influence the future behaviours and attitudes of students. (Karimi, Ashktorab, Mohammadi, & Abedi, 2014; Phillips & Clarke, 2012)

This study has limitations. Study participants were limited to adolescents and young adults studying in vocational school to graduate as practical nurses, who were willing to share their experiences on smoking. The attitudes and beliefs may differ among practical nursing students in Finland and other countries. The findings may also be somewhat different compared to nurse students in a university setting. Furthermore, data were gathered with the first author who had personal and academic history and perspective that may have defined and shaped the phenomena studied. To enhance validity, all quotes from the students were translated by professional Finnish-English translator. Furthermore, our background is both in health sciences and sociology which enabled a broad perspective on study phenomena. The prevalence and knowledge of harms of smokeless tobacco (snuff) and electronic cigarette among healthcare students would be our recommendations as future lines of inquiry. Additionally, longitudinal study setting would give vital information on how knowledge and attitudes of smoking change during education and if smokers identity can be diminished while practical nurses gain professional identity. Implications of this study are not to concentrate on stigmatizing smoking further as it causes rebel and fighting back against the stigma and may make it harder for some to stop smoking (Wigginton & Lee, 2013). Instead of taking to account of the smokers’ identity and enhancing professional identity as a future healthcare worker.

We conclude that practical nursing students who smoked justified their smoking and defended against non-smokers’ contempt in four different ways. The students did not admit smoking hindered their work in the health and social care sector in any way. In personal life and in education, smoking was considered a normal habit of unifying friends and empowering them against life stresses. Strong smoker identity may play a part in preventing students from smoking cessation. Education of healthcare professionals should consider the strong position of smoking in young people’s lives and the strong identity of the student as a smoker. During training, professional identity growth and the ability to override the smoker identity should be invested in the curriculum.
